# Combining an Autophagy Inhibitor, MPT0L145, with Abemaciclib Is a New Therapeutic Strategy in GBM Treatment

**DOI:** 10.3390/cancers13236117

**Published:** 2021-12-04

**Authors:** Tsung-Han Hsieh, Muh-Lii Liang, Jia-Huei Zheng, Yu-Chen Lin, Yu-Chen Yang, Thanh-Hoa Vo, Jing-Ping Liou, Yun Yen, Chun-Han Chen

**Affiliations:** 1 Joint Biobank, Office of Human Research, Taipei Medical University, Taipei 110, Taiwan; thhsieh@tmu.edu.tw (T.-H.H.); wendy31400@tmu.edu.tw (J.-H.Z.); can_0131@tmu.edu.tw (Y.-C.Y.); 2 Neuroscience Research Center, Taipei Medical University Hospital, Taipei 110, Taiwan; 3 Department of Neurosurgery, Mackay Memorial Hospital, Taipei 104, Taiwan; liang4617@hotmail.com; 4 Department of Medicine, Mackay Medical College, New Taipei City 252, Taiwan; 5 Department of Pharmacology, School of Medicine, College of Medicine, Taipei Medical University, Taipei 110, Taiwan; amylin0083@tmu.edu.tw; 6 Graduate Institute of Medical Sciences, College of Medicine, Taipei Medical University, Taipei 110, Taiwan; 7 School of Medicine, Vietnam National University Ho Chi Minh City, Ho Chi Minh City 700000, Vietnam; vthoa@medvnu.edu.vn; 8 School of Pharmacy, College of Pharmacy, Taipei Medical University, Taipei 110, Taiwan; jpl@tmu.edu.tw; 9 The Ph.D. Program for Cancer Molecular Biology and Drug Discovery, College of Medical Science and Technology, Taipei Medical University, Taipei 110, Taiwan; 10 TMU Research Center of Cancer Translational Medicine, Taipei Medical University, Taipei 110, Taiwan; 11 Cell Physiology and Molecular Image Research Center, Wan Fang Hospital, Taipei Medical University, Taipei 116, Taiwan

**Keywords:** glioblastoma multiforme, abemaciclib, MPT0L145, synergism

## Abstract

**Simple Summary:**

Glioblastoma multiforme is a common and lethal malignant brain tumor, and occurrence of therapeutic resistance in GBM often causes relapse. Therefore, this study aimed to find a new therapeutic strategy to treat these malignant tumors. In the current study, combined abemaciclib with an autophagy inhibitor, MPT0L145, significantly reduced cell proliferation and elevated intracellular reactive oxygen species (ROS) level. A blood–brain barrier permeability assay also showed that MPT0L145 could penetrate endothelial cells and has a penetration ability similar to that of TMZ. Therefore, this work represents a potential therapeutic strategy for treating the GBM in the future.

**Abstract:**

Glioblastoma multiforme (GBM) is the most malignant brain tumor in the world, only 25% of GBM patients were alive one year after diagnosis. Although Temozolamide combined with radiation therapy more effectively prolonged the survival rate than radiation alone, the overall survival rate is still dismal. Therefore, a new therapeutic strategy is urgently needed. CDK4/6 inhibitors are newly FDA-approved agents to treat HR-positive, HER2-negative advanced, and metastatic breast cancers, and preclinical results showed that CDK4/6 inhibitors significantly reduced cell proliferation and tumor growth. However, several studies have suggested that CDK4/6 inhibitor-induced non-genetic changes caused treatment failure, including autophagy activation. Therefore, this study aimed to combine an autophagy inhibitor, MPT0L145, with abemaciclib to improve therapeutic efficiency. The use of abemaciclib effectively inhibited cell proliferation via suppression of RB phosphorylation and induced autophagy activation in GBM cancer cells. MPT0L145 treatment alone not only blocked autophagy activation, but also induced generation of ROS and DNA damage in a concentration-dependent manner. Importantly, MPT0L145 had a comparable penetration ability to TMZ in our blood brain barrier permeability assay. Combined MPT0L145 with abemaciclib significantly reduced cell proliferation, suppressed RB phosphorylation, and increased ROS production. In conclusion, the data suggested that blocking autophagy by MPT0L145 synergistically sensitized GBM cancer cells to abemaciclib and represents a potential therapeutic strategy for treating GBM in the future.

## 1. Introduction

Glioblastoma multiforme (GBM) is a common and lethal malignant brain tumor. Current therapeutic strategies include surgery, radiation therapy, chemotherapy, and combined therapy [1,2]. Temozolamide (TMZ) is a DNA alkylating reagent which targets guanine at a position of O^6^ and N^7^. Due to its smaller molecular weight, TMZ is easily able to cross the blood-brain barrier (BBB) [3]. In the past 20 years, TMZ became the first-line chemotherapy to treat GBM, and TMZ plus radiation therapy effectively improves survival rates when compared with radiation therapy alone (14.6 months versus 12.1 months), and increases the 2-year survival rate from 10.4% to 26.5% [4]. However, the occurrence of therapeutic resistance in GBM causes tumor relapse, resulting in a 5-year survival rate of only about 5% [2].

In the past few years, three CDK4/6 inhibitors, namely palbociclib, ribociclib, and abemaciclib, have been approved to treat patients with HR-positive, HER2-negative advanced, and metastatic breast cancers by the U.S. Food and Drug Administration (FDA) [5]. Previously, a comprehensive analysis study showed that alteration of the RB signaling pathway was found in 78% of GBMs, including homozygous deletion of CDKN2A (52%), CDKN2B (47%) and CDKN2C (2%), homozygous deletion or mutation of RB (11%), and amplification of CDK4 (18%), CCND2 (2%) and CDK6 (1%) [6]. Furthermore, a previous study also showed that palbociclib and abemaciclib have the ability to cross the BBB [7]. These data indicate that CDK4/6 inhibitors are potential drugs to treat brain tumors. Recent studies have demonstrated that CDK4/6 inhibitors effectively reduced cell proliferation and tumor growth in several brain tumors, including GBMs [8,9,10,11], AT/RTs [12], medulloblastomas [13,14] and ependymomas [15]. Although treatment with CDK4/6 inhibitors alone can delay tumor growth, some evidence demonstrated that CDK4/6 inhibitors induce nongenetic changes resulting in therapeutic failure, such as activation of the PI3K–AKT–mTOR signaling pathway, activation of cyclin E-CDK2 pathway and occurrence of autophagy [16,17]. Therefore, combining other therapeutic strategies with CDK4/6 inhibitors will be essential to overcome therapeutic resistance. Previous studies showed that combinations of radiation [9] or targeted agents, everolimus [10] and altiratinib [11], with CDK4/6 inhibitors have synergistic effects against GBM cells and tumor growth in vitro and in vivo.

Autophagy is a self-degradative system to maintain cellular homeostasis and ensure cell growth when cells encounter stress [18]. Tumor cells often develop resistance to chemotherapy and radiation therapy through autophagy activation, indicating that it plays a protective role against cytotoxic treatment [19]. CDK4/6 inhibitors have been reported to induce autophagy activation [20,21], and combined with autophagy inhibitors decreased cell proliferation compared to CDK4/6 inhibitor treatment alone [21,22]. In this study, we found that abemaciclib treatment reduced cell proliferation and induced autophagy activation in GBM cells. We combined abemaciclib with an autophagy inhibitor, MPT0L145, and significantly reduced cell proliferation and elevated intracellular reactive oxygen species (ROS) levels. A blood–brain barrier permeability assay also showed that MPT0L145 could penetrate endothelial cells and has a penetration ability similar to that of TMZ. In conclusion, this study provides a rational combination strategy by targeting autophagy against abemaciclib-induced autophagy activation for GBM therapy.

## 2. Materials and Methods

### 2.1. Cell Culture, Reagents and Plasmid

Human GBM cancer cells, U87MG and T98G cells were obtained from Yen Yun’s laboratory [23] and maintained in Minimum Essential Medium (MEM) (Gibco/Life Technologies, Carlsbad, CA, USA) supplemented with 10% fetal bovine serum (Gibco/Life Technologies). M059K cells were obtained from Dr. Kwan-Hwa Chi’s laboratory [24] and maintained in a 1:1 mixture of Dulbecco’s Modified Eagle’s Medium (DMEM) (Gibco/Life Technologies) and Ham’s F12 medium (Gibco/Life Technologies) supplemented with 2.5 mM L-glutamine, 0.5 mM sodium pyruvate, 0.05 mM non-essential amino acids and 10% fetal bovine serum. These cells were incubated at 37 °C in a humidified atmosphere of 5% CO_2_. MPT0L145 was synthesized by Dr. Jing-ping Liou according to previous publications [25]. Abemaciclib was purchased from MedChemExpress (MCE, Monmouth Junction, NJ, USA). For lentiviral expression of shPIK3C3, plasmids containing shRNA was obtained from the RNAi consortium at Academia Sinica, Taiwan.

### 2.2. RNA-Seq Analysis and Reverse Transcription-Quantitative PCR

Total RNA was extracted using TRIZOL reagent (Invitrogen/Life Technologies, Carlsbad, CA, USA) from MPT0L145-treated U87MG cells and control cells, and RNA quality and quantity were assessed using a bioanalyzer and Qubit. The RNA was ligated to an adaptor for further amplification (Illumina^®^ TruSeq^TM^ stranded mRNA, San Diego, CA, USA). All library preparation was performed in the translational core facility of Taipei Medical University. The raw reads files (fastq) were mapped to Hg19 reference by using STAR (v2.6.1), and the count for each gene was calculated using RSEM (v1.3.1). Differential expression genes (DEGs) were identified using the R package (v3.6.1), DESeq2 (v1.26.0), and Ingenuity pathway analysis (IPA, QIAGEN, RedwoodCity, CA, USA) was used to decipher significant regulatory pathways. For reverse transcription-quantitative PCR, the total RNA of drug-treated cells was isolated using the TRIZOL reagent, and reverse-transcribed into complementary DNA through random hexamer priming using a HiScript II Q RT SuperMix (Vazyme). Quantitative PCR was performed in duplicate with gene-specific primers using a Maxima^TM^ SYBR FAST qPCR kit (Thermo Fisher Scientific, Waltham, MA, USA).

### 2.3. Immunoblotting

Immunoblotting was performed using anti-phospho-RB (Cell Signaling, Danvers, MA, USA), anti-p62 (GeneTex, Irvine, CA, USA), anti-LC3B (Novus biologicals, Littleton, CO, USA), anti-caspase3 (Novus biologicals), anti-γH2AX (Cell Signaling), anti-PIK3C3 (GeneTex) and anti-GAPDH (GeneTex) antibodies, followed by visualization using horseradish peroxidase-conjugated secondary antibodies and an enhanced chemiluminescence detection system.

### 2.4. Blood Brain Barrier Permeability In Vitro Assay

Ready-to-use BBB kits (RBT-24H) (PharmaCo-Cell. Nagasaki, Japan) were used. Briefly, the thawed cells, including endothelial cells, brain pericytes and astrocytes were lined on the brain-side chamber and blood-side chamber. The BBB kit was placed in the incubator and re-flash medium every day. After 4 days of incubation, cell growth on the transwell membrane of the micro plate wells and the trans endothelial electrical resistance (TEER) reached more than 150 Ω × cm^2^ to prove the kit was functionally active [26]. Compounds were added into up-side chamber for 4 h. The culture medium in the up-side and down-side were collected independently. The concentrations of the compounds were detected by LC/MS/MS (API2000).

### 2.5. Flow Cytometry for Cell Cycle Analysis and Autophagy

Cells were seeded at a concentration of 1 × 105/well and incubated at 37 °C for 24 h, and then the cells were exposed to abemaciclib or MPT0L145 for 24 and 48 h. The cells were harvested and fixed in cold 70% alcohol overnight at −20 °C, and then stained with propidium iodide (PI) containing 10 mg/mL RNase and analyzed by flow cytometry. For autophagy detection, cells were seeded at a concentration of 1 × 105/well and treated with DAPGreen (Dojindo Molecular Technologies, Rockville, MD, USA) at 37 °C for 30 min, followed by removal of the the supernatant and addition of the drug-containing media. After incubation for 24 h, cells were harvested and detected by fluorescence using flow cytometry. The results were processed using FlowJo.

### 2.6. MTT Assay, LDH Assay and ROS-Glo^TM^ H_2_O_2_ Assay

Cells were seeded at a concentration of 3 × 10^3^/well and incubated at 37 °C for 24 h, and then the cells were exposed to abemaciclib or MPT0L145. After incubation for 72 h, the cells were treated with 1% thiazolyl blue tetrazolium for 1 h at 37 °C followed by the addition of DMSO, and were mixed thoroughly. The results were obtained by measuring the absorbance at 570 and 650 nm using a multi-well scanning spectrophotometer. For the drug interaction study, the combination index (CI) value was generated using the fraction-affected value of each combination according to the Chou–Talalay method, using CompuSyn software (ComboSyn, Inc., Paramus, NJ, USA) [27]. A combination index value below 1 represented synergism. For the LDH assay, cells were seeded at a concentration of 3 × 10^3^/well and treated with drugs at the indicated concentration for 72 h. The culture media was collected and LDH release was measured using the CytoTox 96 Non-Radioactive Cytotoxicity Assay Kit (Promega, Madison, WI, USA) according to the manufacturer’s protocol. For detecting ROS level, cells were seeded at a concentration of 3 × 10^3^/well and treated with drugs at the indicated concentration for 72 h, the H_2_O_2_ substrate was then added and the mixture was incubated at 37 °C. After incubation for 6 h, ROS-Glo^TM^ detecting solution was added and incubated for twenty minutes at room temperature, and then luminescence units were detected using a luminometer.

### 2.7. Statistical Analysis

For the experiments, all data are reported as mean ± standard deviation and analyzed using the two-tailed student’s *t*-test. The differences were considered significant at *p* values less than 0.05.

## 3. Results

### 3.1. Abemaciclib Treatment Reduced Cell Proliferation and Induced Autophagy Activation in GBM Cells

**Figure 1:**
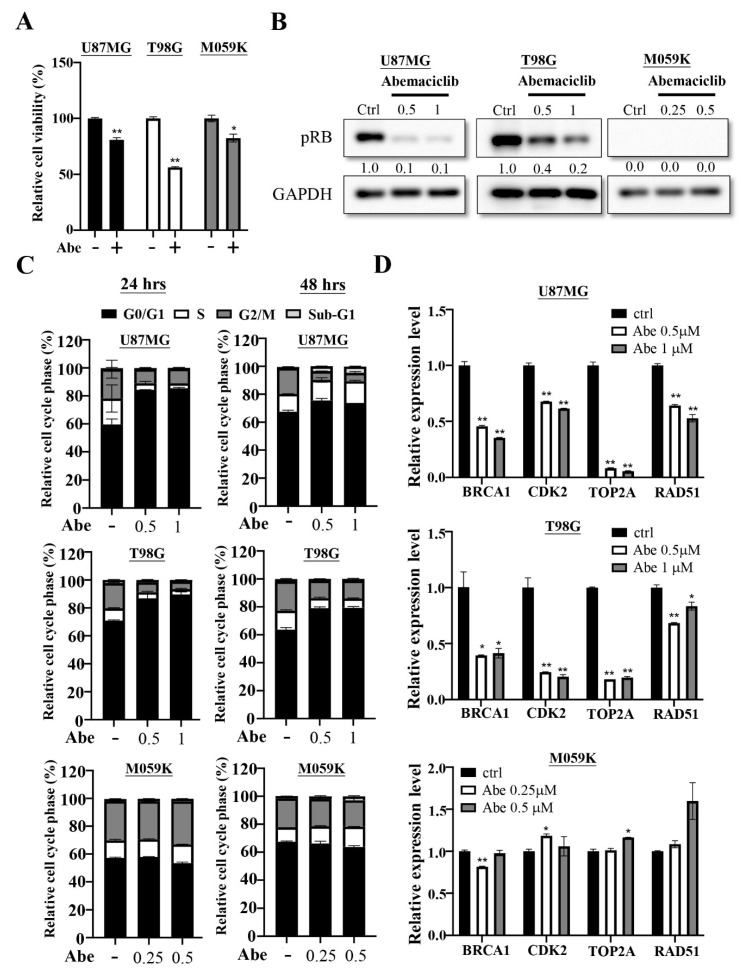
Abemaciclib treatment regulates cell proliferation on the U87MG, T98G and M059K cells. (**A**) The three cancer cells were treated with abemaciclib for 72 h and the proliferation rate was evaluated by using MTT assay. Data are presented as the mean ± SD of duplicate wells, and are representative of two independent experiments. Abe: abemaciclib. (* *p* < 0.05, ** *p* < 0.01 compared to control group) (**B**) The three cancer cells were treated with the indicated concentrations (μM) of abemaciclib for 24 h and the levels of phosphorylated RB were detected via Western blot analysis (Figure S1). (**C**) The three cancer cells were exposed to the indicated concentrations (μM) of abemaciclib for 24 and 48 h and then subjected to cell cycle analysis. Data are presented as the mean ± SD (n = 2). (**D**) Expressions of indicated genes in the three cancer cells were validated by qPCR. The results are presented as the mean ± SD for duplicate samples. (* *p* < 0.05, ** *p* < 0.01 compared to control group).

The comprehensive genomic analysis showed that genomic alteration in RB signaling observed in 78% GBM patients [6], suggesting that CDK4/6 inhibitors were potential therapeutic agents against this disease. We first evaluated the effects of abemaciclib treatment in three GBM cell lines, U87MG, T98G, and M059K. We treated three cancer cells with abemaciclib and determined their proliferation rate using an MTT assay, the result showed that the proliferation rate was decreased compared with the control cells (Figure 1A). We further examined the phosphorylation status of RB, a downstream target of CDK4/6. We treated the cancer cells with different concentrations of abemaciclib for 24 h and found that the phosphorylation levels of RB (Ser807/811) were significantly decreased in U87MG and T98G; however, the M059K showed no signal in RB because it is an RB-deficient cancer cell line (Figure 1B). The cell cycle analysis revealed that treatment with abemaciclib increased the accumulation of cells at the G1 phase at 24 and 48 h in U87MG and T98G, but abemaciclib treatment did not affect the cell cycle fraction in M059K (Figure 1C). Phosphorylation of the RB protein causes E2F protein to dissociate from it, which translocates to the nucleus and regulates gene expression [28]. The E2F-regulated genes are involved in several cellular processes, including the cell cycle, DNA repair, apoptosis and differentiation [29]. Therefore, we verified the expression of multiple genes using qPCR, and the results demonstrated that the mRNA levels of BRCA1, CDK2, TOP2A and RAD51 were decreased after abemaciclib treatment in U87MG and T98G; however, there was no decrease in the mRNA levels in these genes in M059K (Figure 1D). These data indicated that abemaciclib treatment could reduce cell proliferation and alter gene expression in RB-proficient GBM cells. Moreover, abemaciclib treatment also delayed cell proliferation in a cell cycle-independent manner in RB-deficient M059K cells.

**Figure 2:**
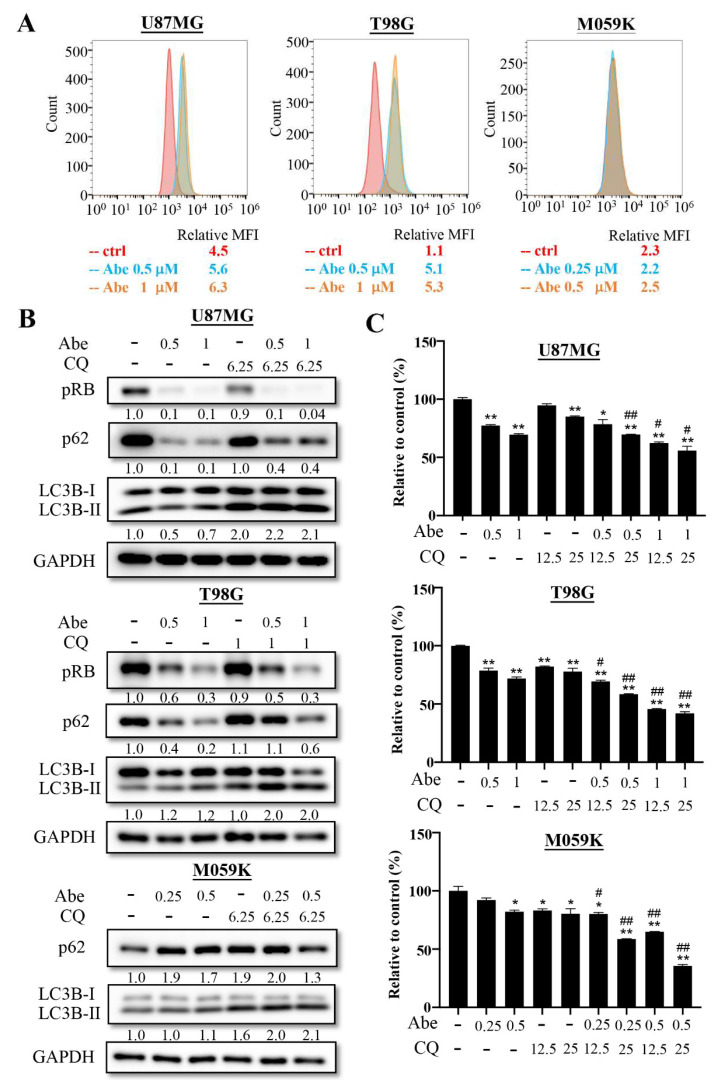
Abemaciclib treatment induces autophagy activation in GBM cells. (**A**) The three cancer cells were treated with control or the indicated concentrations of abemaciclib for 24 h, and autophagy was determined using the DAPGreen Detection Reagent and flow cytometry. The mean fluorescence intensity (MFI) value was relative to the unstained control. (**B**) LC3B-II intensity after abemaciclib treatment (Abe, μM) in the absence or presence of chloroquine (CQ, μM) was detected via Western blot analysis (Figure S2). (**C**) The three cancer cells were treated with indicated concentrations (μM) of abemaciclib in the absence or presence of chloroquine (μM) for 72 h and subjected to MTT assay. Data are presented as the mean ± SD of duplicate wells and are representative of three independent experiments. Abe: abemaciclib. CQ: chloroquine. (* *p* < 0.05, ** *p* < 0.01 compared to control group, # *p* < 0.05, ## *p* < 0.01 compared to indicated concentrations of abemaciclib).

Previous studies have indicated that autophagy is a stress response to cell cycle arrest [21], and previous studies have demonstrated that CDK4/6 inhibitor treatment could induce autophagy activation [20,21,22]. Therefore, we investigated the effects of abemaciclib on autophagy using flow cytometry in GBM cancer cells. DAPgreen is a small florescent molecule which can be incorporated into the autophagosome and is detected by flow cytometry. The results showed that the levels of autophagy were upregulated under abemaciclib treatment in U87MG and T98G cells. Autophagy activation was not observed in M059K cells, since abemaciclib treatment did not cause cell cycle arrest (Figure 2A). We also confirmed the effect of abemaciclib on autophagic flux via Western blotting to detect the expression of LC3B and the autophagy-specific substrate, p62 (Figure 2B). In U87MG and T98 cells, abemaciclib increased the levels of LC3B-II under conditions where degradation of the autophagosomes was blocked by chloroquine, suggesting a promotional effect on the rate of autophagosome formation. We also noticed that abemaciclib itself decreased LC3B-II levels in U87MG cells, which may have resulted from extensive degradation of the autophagosomes. Meanwhile, abemaciclib strongly decreased the levels of p62 in the U87MG and T98 cells, indicating complete autophagy upon drug treatment. However, we could not find the same phenomenon in the M059K cells, suggesting that abemaciclib is not able to induce autophagy in Rb-deficient cells. To determine whether pharmacological inhibition of autophagy could synergize with abemaciclib to reduce cell proliferation, we combined chloroquine with abemaciclib and detected the proliferation rate using an MTT assay (Figure 2C). We found that chloroquine could further reduce cell proliferation with abemaciclib, suggesting that inhibition of autophagy significantly enhances abemaciclib’s ability.

### 3.2. MPT0L145 Is an Autophagy Inhibitor and Could Block Autophagy Process in GBM Cells

**Figure 3:**
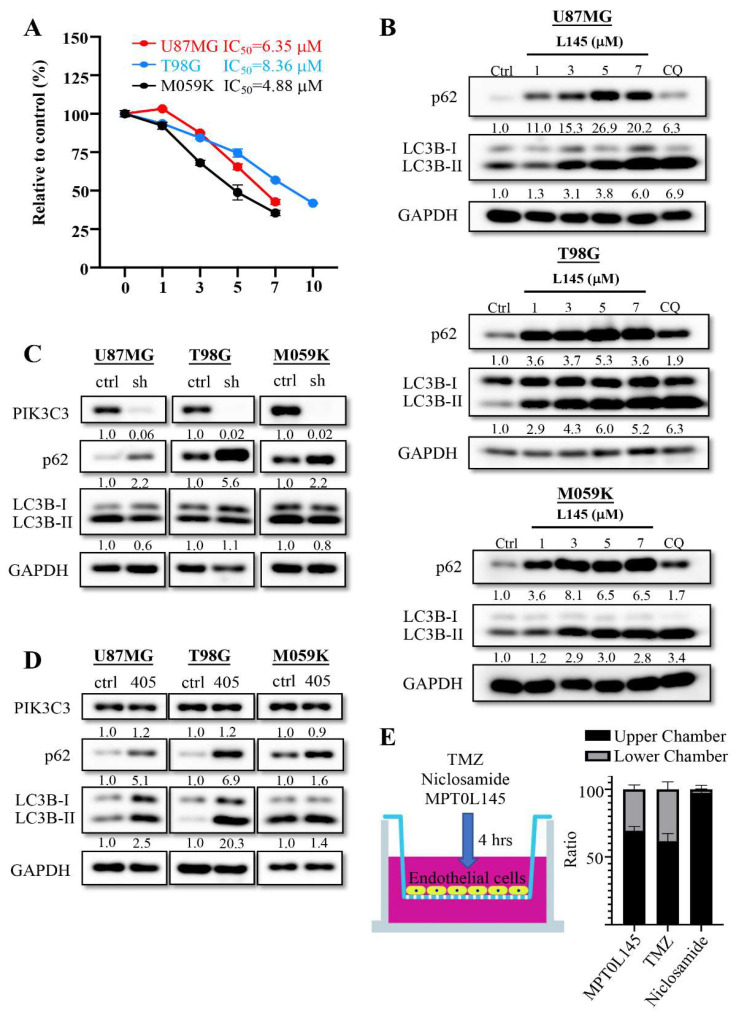
MPT0L145 increases incomplete autophagy in U87MG, T98G and M059K cells. (**A**) The three cancer cells were treated with MPT0L145 for 72 h and the IC_50_ value was evaluated using MTT assay. Data are presented as the mean ± SD of triplicate wells, and are representative of two independent experiments. (**B**) The three cancer cells were exposed to the indicated concentrations (μM) of MPT0L145 and chloroquine (12.5 μM) for 24 h. The protein lysates were subjected to Western blot analysis with the indicated antibodies (Figure S3). (**C**) PIK3C3 was stably depleted in the three cancer cells and the levels of indicated proteins were detected via Western blot analysis (Figure S3). (**D**) The three cancer cells were exposed to the PIK3C3 inhibitor SAR405 (10 μM), for 24 h. Protein lysates were subjected to Western blot analysis with the indicated antibodies (Figure S3). (**E**) TMZ, Niclosamide and MPT0L145 were injected into the upper chamber of the ready-to-use BBB kit for 4 h. The supernatants of the upper and lower chambers were collected and analyzed by LC/MS/MS. The ratio was calculated as the concentration of indicated chamber/(concentration of lower chamber plus concentration of upper chamber) × 100% and the data are presented as the mean ± SD of four trans wells.

MPT0L145 is a PIK3C3/FGFR3 inhibitor which simultaneously promotes autophagosome formation via FGFR3 inhibition and impairs autophagy flux via PIK3C3 inhibition in bladder cancer cells with FGFR3 activation [25,30]. Combined with MPT0L145, it could synergistically sensitize A549 and Panc-1 cells to gefitinib and gemcitabine, respectively [31]. To investigate the effects of MPT0L145 on GBM cancer cells, we treated cells with different concentrations of MPT0L145 and calculated their IC_50_ values using an MTT assay (Figure 3A). The IC50 values of MPT0L145 in T98G, U87MG and M059K cells were 8.36, 6.35 and 4.88 μM, respectively. To realize whether MPT0L145 affected the autophagy process, LC3B and the autophagy-specific substrate (p62) were examined using Western blotting. The results showed that the incubation of different concentrations of MPT0L145 for 24 h induced an increase in LC3B-II and increased the levels of p62 compared to the control cells (Figure 3B). The data suggested MPT0L145 triggered incomplete autophagy in GBM cells, which was also found when the cells were exposed to an autophagy inhibitor (chloroquine). We further checked whether inhibition of PIK3C3 could regulate the autophagy process in GBM cancer cells; thus, we knocked down PIK3C3 to mimic the function of MPT0L145 using the PIK3C3 inhibitor, SAR405, and checked the levels of p62 and LC3B. Knocking down PIK3C3 expression enhanced the levels of p62, but this was not the case in LC3B-II expression (Figure 3C). However, SAR405 treatment simultaneously enhanced p62 and LC3B-II expression levels (Figure 3D). These data demonstrated that MPT0L145 could block the autophagy process through targeting PIK3C3 and thus affecting p62 degradation, suggesting that MPT0L145 has similar function to chloroquine in GBM cancer cells. The ability to pass the blood–brain barrier is an important issue for evaluating whether the developed drugs could be used to treat brain tumors. Thus, we loaded MPT0L145 into the upper chamber of a ready-to-use blood–brain barrier in vitro assay kit for 4 h. In this study, we also loaded TMZ and Niclosamide as positive and negative controls, respectively. After incubation for 4 h, the supernatants of the upper and lower chamber were collected and the concentration of drugs was detected using LC/MS/MS (Figure 3E). The results showed that MPT0L145 could be detected in the upper and lower chamber like TMZ. However, Niclosamide was only detected in the upper chamber. This data suggested that MPT0L145 could pass the blood brain barrier, and have similar abilities as TMZ.

### 3.3. MPT0L145 Treatment Regulates Gene Expression and Promotes ROS Generation and DNA Damage

**Figure 4:**
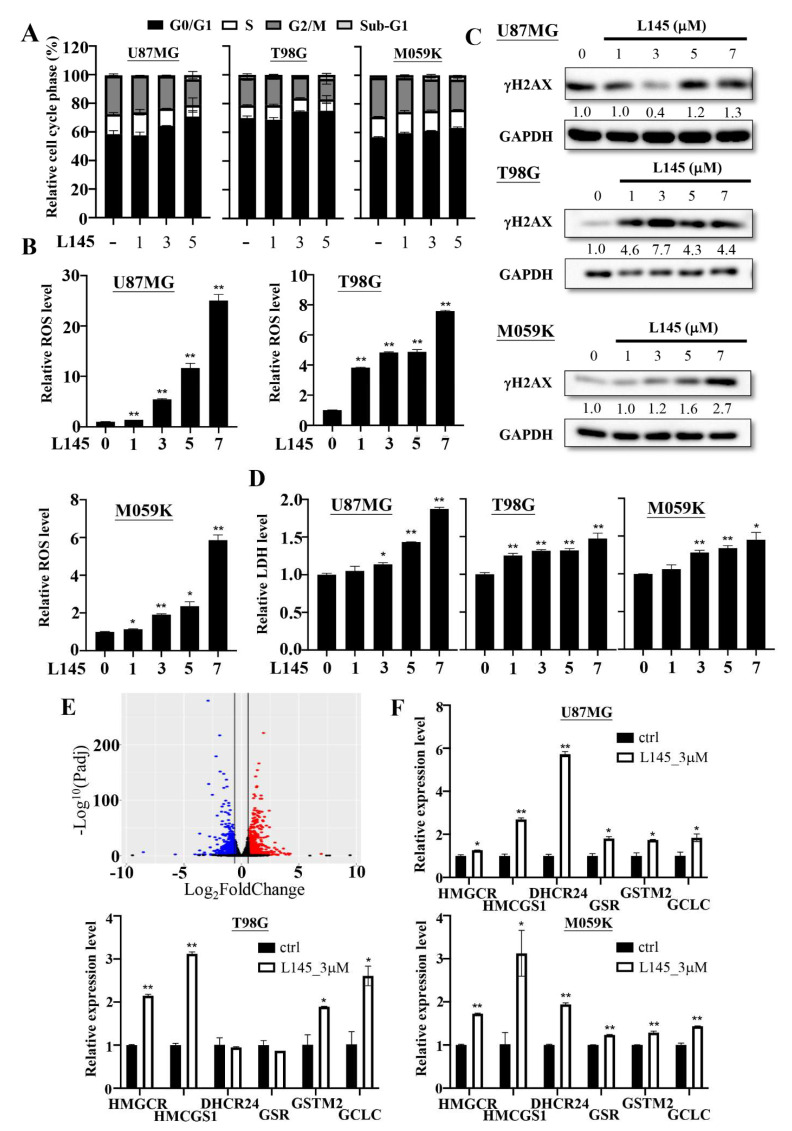
Effects of MPT0L145 on the U87MG, T98G and M059K cells. (**A**) The three cancer cells were exposed to the indicated concentrations (μM) of MPT0L145 for 24 h and then subjected to cell cycle analysis. Data are presented as the mean ± SD (n = 2). (**B**) The three cancer cells were treated with the indicated concentrations (μM) of MPT0L145 for 72 h. Accumulation of intracellular ROS levels were measured by luminometer. The luminescence units were normalized by the absorption value (O.D. 570 nm) from the MTT assay representing cell amounts, and fold changes were calculated and presented as the mean ± SD. (* *p* < 0.05, ** *p* < 0.01 compared to control group) (**C**) The three cancer cells were incubated with the indicated concentrations of MPT0L145 for 72 h. Protein lysates were subjected to Western blot analysis for detection of the DNA damage marker, γH2AX (Figure S4). (**D**) The three cancer cells were treated with the indicated concentrations (μM) of MPT0L145 for 72 h. The supernatant was collected and subjected to LDH analysis. Data are presented as the mean ± SD. (* *p* < 0.05, ** *p* < 0.01 compared to control group) (**E**) A volcano plot showed significantly upregulated (red) and downregulated (blue) genes after MPT0L145 treatment (3 μM) for 48 h in U87MG cells. (**F**) Expressions of the indicated genes in the three cancer cells were validated by qPCR. The results are presented as the mean ± SD for duplicate samples. (* *p* < 0.05, ** *p* < 0.01 compared to control group).

MPT0L145 treatment reportedly arrested the cell cycle in the G1 phase and contributed to cell death via ROS accumulation and DNA damage in bladder cancers [25,30]. Therefore, we examined the effects of MPT0L145 treatment on cell cycle progression in GBM cancer cells. The data revealed that treatment with MPT0L145 increased the accumulation of GBM cells in the G1 phase at 24 h (Figure 4A). We further checked whether MPT0L145 treatment contributed to ROS production and DNA damage. The data showed that the generation of ROS and the levels of the DNA damage marker γH2AX, were increased in a concentration-dependent manner over 72 h (Figure 4B,C). To verify whether MPT0L145 treatment contributed to cell death, we treated it with different concentrations of MPT0L145 for 72 h and determined the cleavage of caspase-3 where paclitaxel was included as a positive control. We found that MPT0L145 treatment was not able to increase the cleavage of caspase-3 (Figure S5). A previous study reported that ROS could promote necroptosis, a type of programmed cell death [32]; thus, the MPT0L145-treated culture media were used to detect the levels of LDH. The data showed that the levels of LDH were increased in a concentration-dependent manner over 72 h in GBM cancer cells (Figure 4D). These data indicated that MPT0L145 caused the antitumor activity in GBMs.

To elucidate the mechanisms underlying the anticancer activity of MPT0L145, mRNA sequencing of U87MG cells treated with MPT0L145 or vehicle control for 48 h was performed, and alterations in global expression were analyzed. The volcano plot showed that a total of 959 genes had increased and 700 genes had decreased expression after MPT0L145 treatment (Padj < 0.05, fold change ≥ 1.5) (Figure 4E). Ingenuity pathway analysis (IPA) was performed to find the significant differential pathways (Table S1). Interestingly, cyclins and the cell cycle regulation and oxidative stress response were significantly down-regulated and up-regulated in MPT0L145-treated cells, respectively. These data were correlated with the anticancer activity of MPT0L145, which are reported in Figure 4A,B. Moreover, cholesterol biosynthesis was also significantly up-regulated in the MPT0L145-treated cells. We further verified the expressions of the oxidative stress-responsive genes and the cholesterol biosynthesis-related genes using qPCR in the GBM cancer cells and recorded an increase in the mRNA levels of HMGCR, HMCGS1, DHCR24, GSR, GSTM2 and GCLC after MPT0L145 treatment in U87MG and M059K cells (Figure 4F). However, the DHCR24 and GSR genes were not up-regulated in MPT0L145-treated T98G cells.

### 3.4. Drug Combination of Abemaciclib and MPT0L145 Treatment Provides Synergistic Anti-Proliferation Effects and Increase ROS Level

**Figure 5:**
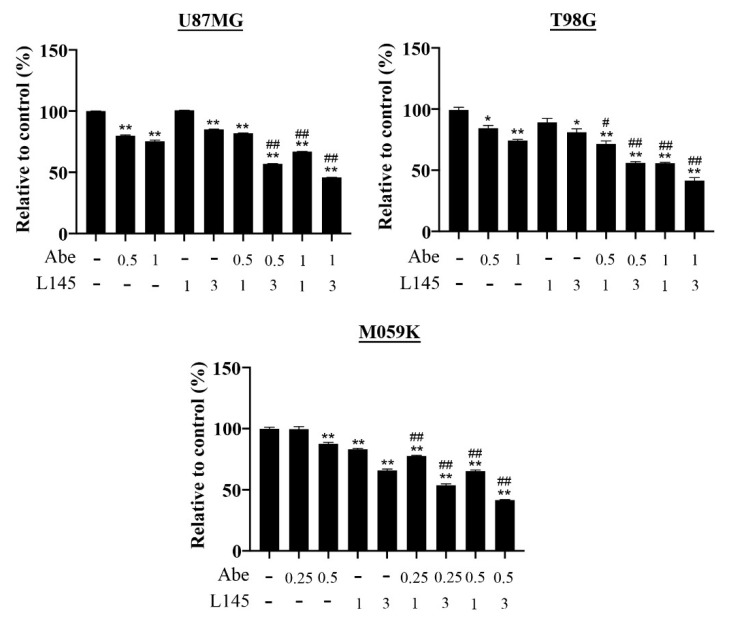
MPT0L145 sensitized cancer cells to abemaciclib. The three cancer cells were treated with the indicated concentrations of abemaciclib in the absence or presence of the indicated concentrations (μM) of MPT0L145 for 72 h and subjected to MTT assay. Data are presented as the mean ± SD of duplicate wells and are representative of three independent experiments. Abe: abemaciclib. L145: MPT0L145. (* *p* < 0.05, ** *p* < 0.01 compared to control group, # *p* < 0.05, ## *p* < 0.01 compared to indicated concentrations of abemaciclib).

**Figure 6:**
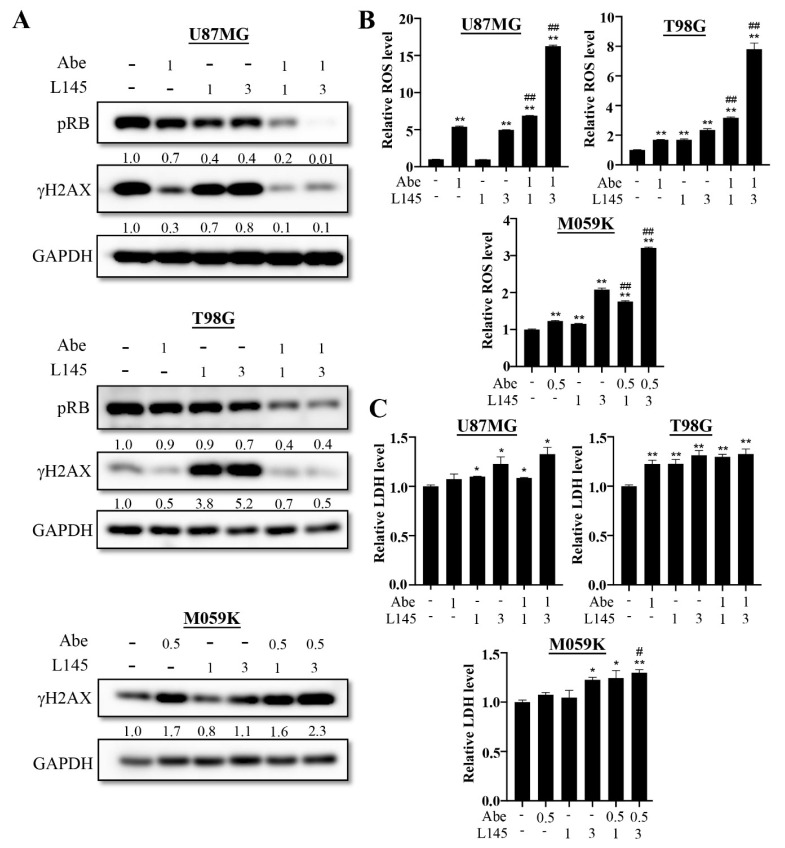
Effects of drug combinations on the U87MG, T98G and M059K cells. (**A**) The three cancer cells were treated with abemaciclib (μM) in the absence or presence of the indicated concentrations of MPT0L145 (μM) for 72 h. Protein lysates were subjected to Western blot analysis for detection of the levels of phosphorylated RB and γH2AX (Figure S6). (**B**) The three cancer cells were treated with abemaciclib (μM) in the absence or presence of indicated concentrations (μM) of MPT0L145 for 72 h. Accumulation of intracellular ROS levels were measured by luminometer. The luminescence units were normalized by the absorption value (O.D. 570 nm) from the MTT assay representing cell amounts, and fold changes were calculated and presented as the mean ± SD. (* *p* < 0.05, ** *p* < 0.01 compared to control group, # *p* < 0.05, ## *p* < 0.01 compared to indicated concentrations of abemaciclib) (**C**) The three cancer cells were exposed to abemaciclib (μM) in the absence or presence of indicated concentrations (μM) of MPT0L145 for 72 h. The supernatant was collected and subjected to LDH analysis. Data are presented as the mean ± SD. (* *p* < 0.05, ** *p* < 0.01 compared to control group, # *p* < 0.05, ## *p* < 0.01 compared to indicated concentrations of abemaciclib).

Previous results showed abemaciclib treatment induces autophagy activation in GBM cancer cells, which led us to hypothesize that targeting autophagy in MPT0L145 might increase its sensitivity. Therefore, we combined MPT0L145 with abemaciclib for 72 h. The data reveal that abemacilib plus MPT0L145 treatment more efficiently reduced cell viability than single agent treatment (Figure 5). The combination index (CI) values showed that 3 μM MPT0L145 synergistically sensitized the three GBM cell lines to abemaciclib (Table S2). To realize the mechanisms underlying the synergetic effects on combined treatment, we first examined whether the effects of drug combination delayed cell proliferation or caused cell death; thus, we combined MPT0L145 with abemaciclib for 72 h, and detected the protein levels of phosphorylated RB and H2AX (Figure 6A). The results showed that combination treatment decreased the levels of phosphorylated RB and γH2AX in both the U87MG and T98G cells. However, the γH2AX signals increased upon the combination treatment in M059K cells. We further determined the cleavage of caspase 3 to determine whether or not the combination treatment induced apoptosis. The results showed that the cleavage of caspase 3 was not observed in the U87MG and T98G cells, but was observed in M059K cells (Figure S7). These results showed that MPT0L145 treatment induced ROS production and promoted LDH release (Figure 4B,D). To determine whether the combination treatment induced ROS production and necroptosis, the GBM cancer cells were treated with MPT0L145 and abemaciclib, and the LDH assay and the ROS-Glo^TM^ H_2_O_2_ assay were used to detect necroptosis and ROS production. The data showed that generation of ROS were observed in the abemaciclib treatment alone, and the combination treatment significantly increased the levels of ROS in three GBM cancer cells (Figure 6B). In the U87MG and T98G cells, when comparing with the control, both abemaciclib and combination treatment moderately increased LDH levels, yet the differences between these treatments were not significant (Figure 6C). On the contrary, in the M059K cells, combination treatment increased LDH levels more than with abemaciclib treatment alone. These results indicated that halting autophagy by MPT0L145 is a promising approach to inhibit cell proliferation in RB-proficient GBM cancer cells, but it induces cell death in RB-deficient M059K cells.

## 4. Discussion

Autophagy is a tightly regulated process of response to various cytotoxic stresses, in which cells degrade their own cellular contents to promote cell survival [33]. The initiation target of autophagy is the Unc-51-like kinase 1 (ULK1) complex, which triggers the nucleation of the phagophore by phosphorylating PI3KC3 complex I, and then activates local phosphatidylinositol-3-phosphate (PI3P) production and induces the autophagy process. Autophagy in GBM plays different roles in various conditions [4]. As a tumor suppressor, autophagy activation could protect genomic integrity by removing damaged organelles and p62-tagged aggregates, which could produce ROS and cause genomic instability, thus inducing tumor progression. As a tumoral promotor, autophagy activation could protect cancer cells from chemotherapeutic agents- or radiation-induced cytotoxicity, resulting in treatment resistance and tumor progression. A previous study also showed that radiation exposure increased p62, LC3B and Beclin-1 expression, and the level of p62 and LC3B is correlated with poor survival in GBM [4]. Therefore, a combined standard treatment in GBM, including TMZ and radiation plus autophagy inhibitors could possibly guard against autophagy-induced therapeutic resistance. Ye et al. showed that combination of radiation treatment and CQ decreased cell proliferation and activated apoptosis [34]. Furthermore, Hsu et al. also showed that combination TMZ and CQ treatments increased apoptotic cells more than TMZ treatment alone [24]. These data suggest that autophagy inhibitors synergistically enhance treatment efficiency.

PI3KC3, also known as vacuolar protein sorting 34 (vps34), belongs to the class III phosphoinositide 3-kinases (PI3Ks), and it is an indispensable kinase in autophagy [35]. Therefore, PIK3C3 as an important target in the development of autophagy inhibitors, and some drugs have been developed to do so, including spautin-1, SAR405, and MPT0L145 [30,36]. MPT0L145 is a dual-target inhibitor against FGFRs and PIK3C3 [25,30]. In our previous study, MPT0L145 arrested cell proliferation through inhibition of the phosphorylation of FGFR1 and FGFR3 in bladder cancer cells. MPT0L145 treatment also caused p62 accumulation and elevated LC3B-II expression by targeting PIK3C3. In addition, inhibition of PIK3C3 also induced ROS production and γH2AX expression, but not cleavage of caspase 3. As an autophagy inhibitor, MPT0L145 was used to combine some chemotherapeutic agents which induced autophagy activation. In our previous preclinical study, combining MPT0L145 with gefitinib and gemcitabine synergistically sensitizes anticancer effects in A549 cells and PANC-1 cells, respectively [31]. In the current study, we found that MPT0L145 treatment caused p62 accumulation, elevated LC3B-II expression, ROS production, and γH2AX expression in GBM cells. It also up-regulated cholesterol biosynthesis-associated genes, which are similar to our findings in bladder cancer cells [25,30]. During autophagy initiation, the isolated membranes are mainly derived from pre-formed organelle membranes, such as ER. However, the phagophore membrane reportedly expands along with localized phospholipid synthesis [37]. Recent studies suggested that cholesterol accumulation reduces the ability of lysosomes to fuse with endocytic and autophagic vesicles [38]. Our previous study also showed that autophagosomes and LysoTracker-labelled lysosomes were surrounding the enlarged late endosomes, and no fusion were observed in MPT0L145-treated RT-112 cells [30]. As cholesterol is crucial to the formation of biological membranes and MPT0L145 increases incomplete autophagy (Figure 3B), it is wort studying whether the induction of cholesterol bio-synthesis is related to the dysregulation of autophagy in GBM cells. Importantly, MPT0L145 had a comparable penetration ability to TMZ in our blood brain barrier permeability assay. These data suggest that MPT0L145 is a potential drug for use in GBM treatment.

Currently, three CDK4/6 inhibitors, palbociclib, ribociclib, and abemaciclib, have been approved by the U.S. FDA for the treatment of HR-positive, HER2-negative advanced, or metastatic breast cancers. Palbociclib and ribociclib have been demonstrated to have greater affinity for CDK4 and CDK6 than other CDKs [39]. Abemaciclib has a multi-target property, targeting not only CDK4 and CDK6, but also CDK9, PIM1, HIPK2, DYRK2, CK2 and GSK3b [40]. In addition to in brain tumors, preclinical studies have been demonstrated that CDK4/6 inhibitors effectively arrest cell cycle progression and tumor growth in several cancers, including hepatocellular carcinoma [41,42], synovial sarcoma [43], gastric cancers [22], non–small cell lung cancer [44], Ewing’s sarcoma [45], multiple myeloma [20], and aggressive germinal center-derived B-cell lymphoma [46]. Although CDK4/6 inhibitors have a powerful ability to inhibit cell growth, there are some genetic or non-genetic changes induced by CDK4/6 inhibitors that affect treatment response, and autophagy is one of the mechanisms to resist CDK4/6 inhibitor treatment [16,17]. Vijayaraghavan et al. demonstrated that palbociclib treatment induced autophagy activation in breast cancer cells, and a combination of autophagy inhibitor, chloroquine (CQ), with palbociclib caused irreversible cell growth inhibition and senescence in vitro [21]. Valenzuela et al. also demonstrated that Spautin-1 blocked palbociclib-induced autophagy activation and synergistically decreased cell numbers in gastric cancer cells [22]. In addition to palbociclib, abemaciclib treatment also induced autophagy activation in multiple myeloma cells [20]. In our results, we demonstrated that abemaciclib treatment inhibited cell proliferation in three GBM cancer cells including RB-deficient M059K cells, which means abemaciclib inhibits cell growth, possibly in an Rb-independent manner. We also noticed that the γH2AX signals increased upon single agent and combination treatment in M059K cells (Figure 6A). A previous study showed that Rb-knockout fibroblasts accumulated replication-dependent DNA-double-strand breaks and elevated γH2AX signals following chemotherapeutic treatment [47]. This may explain the effect of abemaciclib on γH2AX in Rb-deficient M059K cells. Therefore, it is wort studying the relationship between RB status and γH2AX signals in the future. We also found that abemaciclb treatment induced autophagosome formation and autophagy activation in U87MG and T98G cells. Combinations of abemaciclib and CQ or MPT0L145 resulted in a stronger reduction of GBM cancer cell proliferation than abemaciclib treatment alone. These data suggested that a combined treatment of abemaciclib with an autophagy inhibitor is possibly beneficial for the treatment of GBMs.

## 5. Conclusions

In summary, a previous genomic study showed that genetic changes in the components of RB signaling pathway were observed in GBMs. Our data demonstrated that the CDK4/6 inhibitor abemaciclib effectively inhibited cell proliferation and activated autophagy. Combining an autophagy inhibitor, MPT0L145, with abemaciclib synergistically decreased cell proliferation. Moreover, MPT0L145 treatment alone also provides significant anti-tumor activity. Finally, our preclinical data supported that abemaciclib plus MPT0L145 as a potential strategy to treat GBM in the future.

## Data Availability

The data presented in this study are available in this article (and Supplementary Material).

## Supplementary Materials

The following are available online at https://www.mdpi.com/article/10.3390/cancers13236117/s1, Table S1. Significantly pathways analyzed by IPA. Table S2. The combination index (CI) values of different drug combinations in GBM cancer cells. Figure S1: Abemaciclib treatment regu-lates RB phosphorylation on the U87MG, T98G and M059K cells. Figure S2: Abemaciclib treat-ment induces autophagy activation in GBM cells. Figure S3: MPT0L145 targets PIK3C3 and in-creases incomplete autophagy on U87MG, T98G and M059K cells. Figure S4: MPT0L145 treat-ment induces up-regulation of DNA damage marker, γH2AX, in GBM cells. Figure S5: Effects of MPT0L145 on apoptosis. The three cancer cells were treated with indicated concentrations of MPT0L145 for 72 h. Protein lysates were subjected to Western blot analysis for detection cleavage fragments of caspase 3. Figure S6: Status of RB and γH2AX after combinations treatment on the U87MG, T98G and M059K cells. Figure S7: Effects of drug combination on apoptosis. The three cancer cells were treated with abemaciclib in the absence or presence of indicated concentrations of MPT0L145 for 72 h. Protein lysates were subjected to Western blot analysis for detection cleav-age fragments of caspase 3.
