# Supplementary material for: Combining an Autophagy Inhibitor, MPT0L145, with Abemaciclib Is a New Therapeutic Strategy in GBM Treatment
Source: Cancers (Basel). 2021 Dec 4;13(23):6117. doi: 10.3390/cancers13236117 (PMC8656550; doi:10.3390/cancers13236117)
Supplement: Supplementary file 1 [file cancers-13-06117-s001.zip › cancers-1454656-supplementary.pdf]

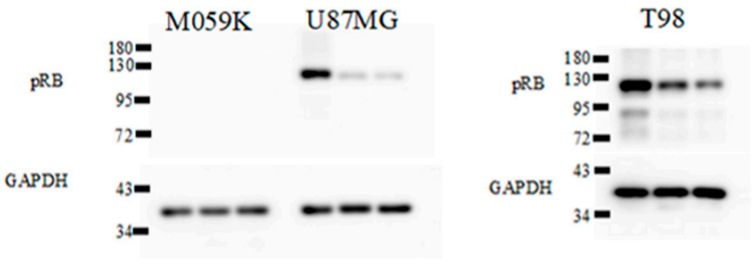

Figure S1. Abemaciclib treatment regulates RB phosphorylation on the U87MG, T98G and M059K cells.

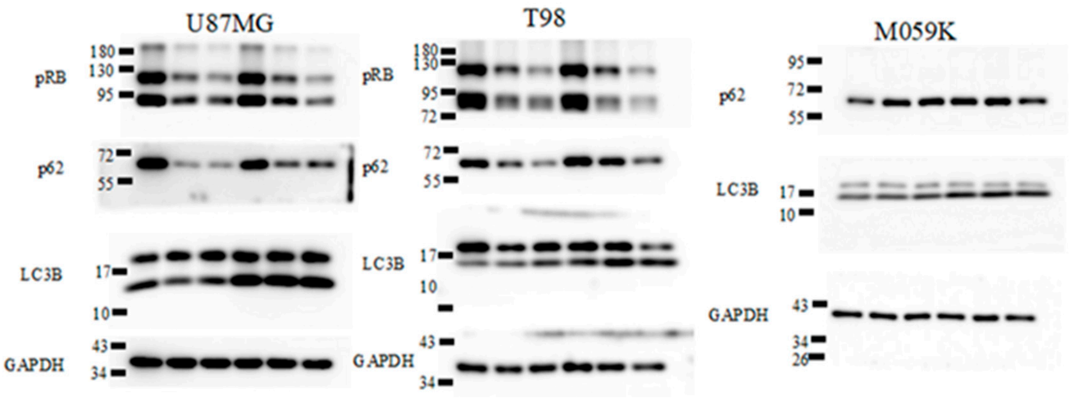

Figure S2. Abemaciclib treatment induces autophagy activation in GBM cells.

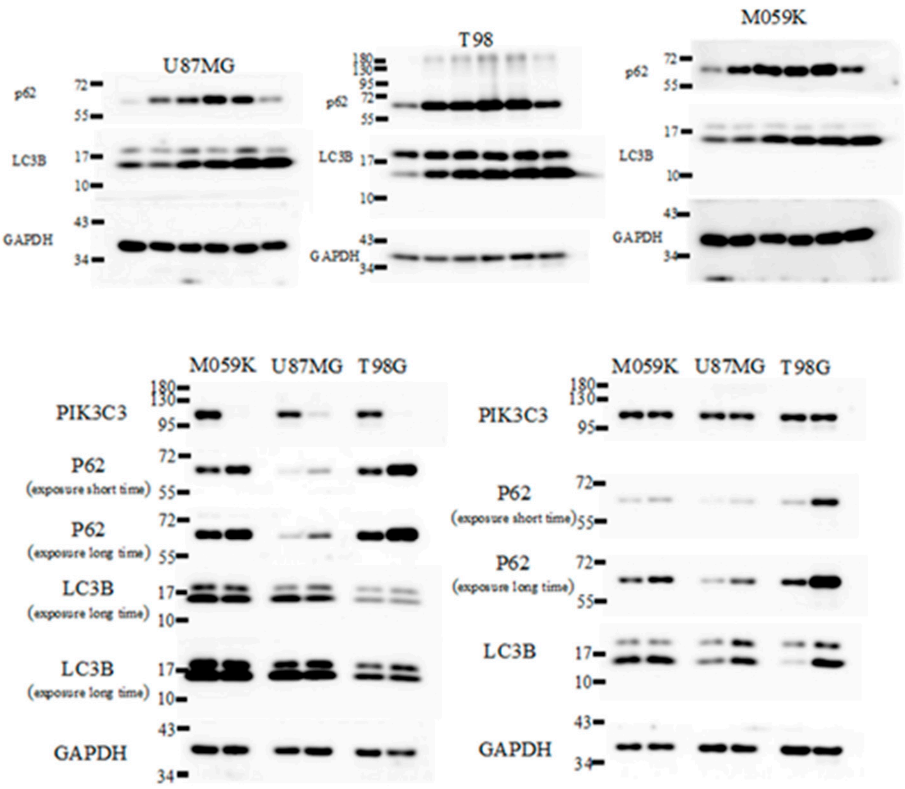

Figure S3. MPT0L145 targets PIK3C3 and increases incomplete autophagy on U87MG, T98G and M059K cells.

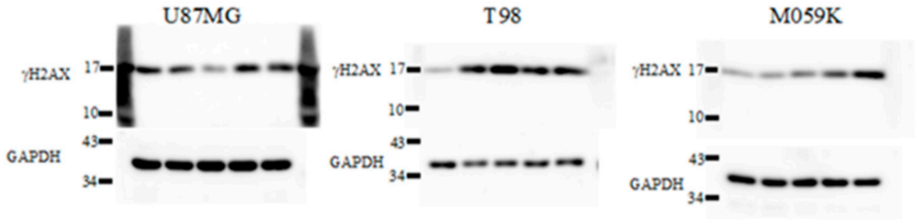

Figure S4. MPT0L145 treatment induces up-regulation of DNA damage marker, γH2AX, in GBM cells.

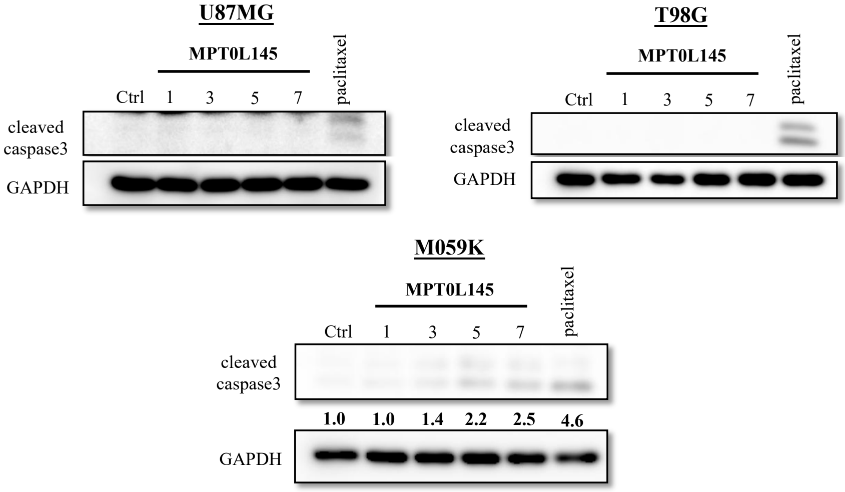

Figure S5. Effects of MPT0L145 on apoptosis. The three cancer cells were treated with indicated concentrations of MPT0L145 for 72 h. Protein lysates were subjected to Western blot analysis for detection cleavage fragments of caspase 3.

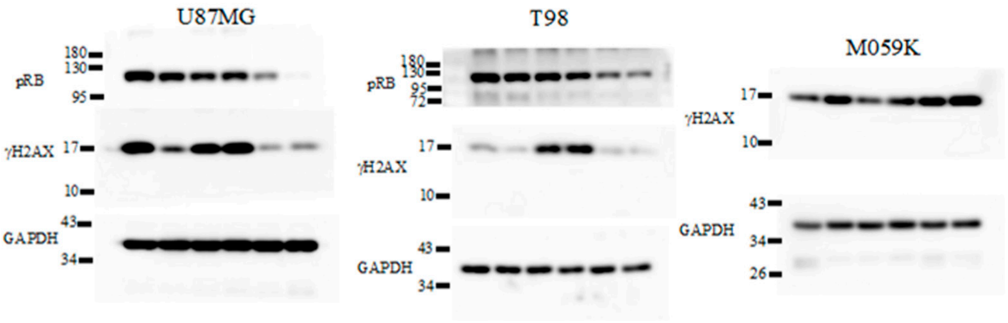

Figure S6. Status of RB and γH2AX after combinations treatment on the U87MG, T98G and M059K cells.

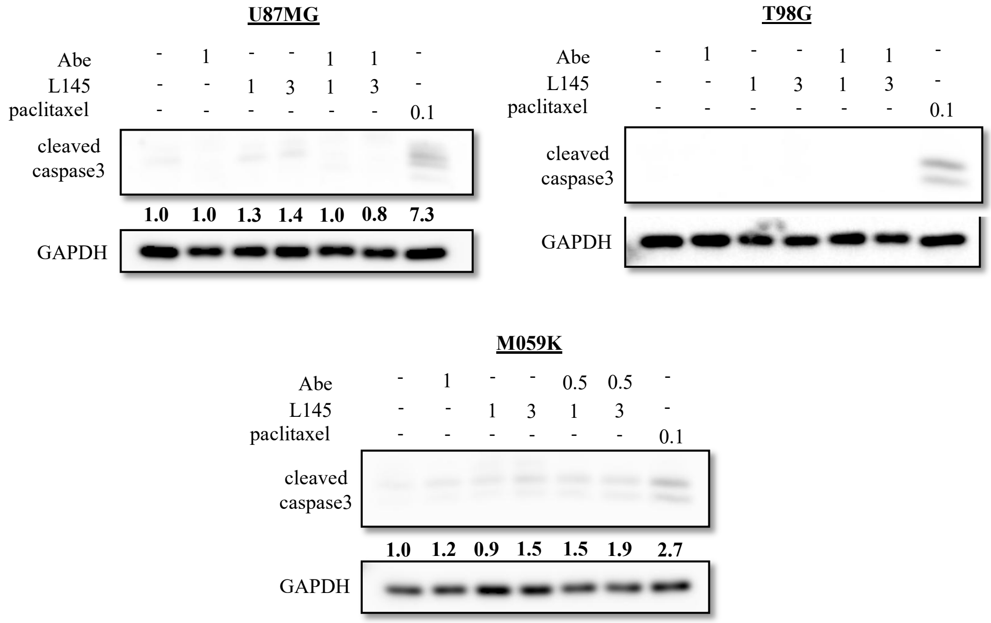

Figure S7. Effects of drug combination on apoptosis. The three cancer cells were treated with abemaciclib in the absence or presence of indicated concentrations of MPT0L145 for 72 h. Protein lysates were subjected to Western blot analysis for detection cleavage fragments of caspase 3.

Table S1. Significantly pathways analyzed by IPA

Up-regulated Pathways

Ingenuity Canonical Pathways	-log(p-value)	Ratio	z-score
PTEN Signaling	1.58	0.127	2.84
Superpathway of Cholesterol Biosynthesis	5.76	0.407	2.714
Mevalonate Pathway I	2.92	0.417	2.236
Superpathway of Geranylgeranyldiphosphate Biosynthesis I (via Mevalonate)	2.29	0.312	2.236
Phospholipases	1.6	0.158	2.121
PPARα/RXRα Activation	2.43	0.135	2.065
Oleate Biosynthesis II (Animals)	2.34	0.4	2
Methylglyoxal Degradation III	1.89	0.308	2
Endothelin-1 Signaling	3.33	0.149	1.8
PEDF Signaling	1.72	0.146	1.732
Cell Cycle: G2/M DNA Damage Checkpoint Regulation	2.01	0.184	1.667
Cholesterol Biosynthesis I	3.72	0.462	1.633
Cholesterol Biosynthesis II (via 24,25-dihydrolanosterol)	3.72	0.462	1.633
Cholesterol Biosynthesis III (via Desmosterol)	3.72	0.462	1.633
Superpathway of Inositol Phosphate Compounds	2.64	0.136	1.569
JAK/Stat Signaling	1.44	0.138	1.508
Gαq Signaling	4.21	0.171	1.46
3-phosphoinositide Biosynthesis	2.41	0.138	1.279
UVA-Induced MAPK Signaling	2.65	0.165	1.265
SPINK1 General Cancer Pathway	1.83	0.164	1.265
Apelin Pancreas Signaling Pathway	1.4	0.163	1.134
D-myo-inositol-5-phosphate Metabolism	2.39	0.14	1.091
Adrenomedullin signaling pathway	1.76	0.12	1.091
Th1 Pathway	2.2	0.145	1.069
PPAR Signaling	1.73	0.139	1.069
Pentose Phosphate Pathway	2.34	0.4	1
NAD biosynthesis II (from tryptophan)	2.02	0.333	1
Superpathway of Citrulline Metabolism	1.66	0.267	1
Oxidative Ethanol Degradation III	1.66	0.267	1
Ethanol Degradation IV	1.31	0.211	1

Down-regulated Pathways

Ingenuity Canonical Pathways	-log(p-value)	Ratio	z-score
ERK/MAPK Signaling	2.31	0.13	-1
Adenosine Nucleotides Degradation II	1.66	0.267	-1
Fatty Acid α-oxidation	1.56	0.25	-1
Purine Nucleotides Degradation II (Aerobic)	1.38	0.222	-1
α-Adrenergic Signaling	1.35	0.129	-1
ErbB Signaling	2.03	0.151	-1.069
Actin Cytoskeleton Signaling	1.3	0.108	-1.147
Glioma Signaling	2.92	0.165	-1.155
Fcγ Receptor-mediated Phagocytosis in Macrophages and Monocytes	2.03	0.151	-1.387
Actin Nucleation by ARP-WASP Complex	1.45	0.143	-1.414
T Cell Exhaustion Signaling Pathway	1.3	0.112	-1.5
PD-1, PD-L1 cancer immunotherapy pathway	1.35	0.126	-1.508
Estrogen-mediated S-phase Entry	1.96	0.231	-1.633
Inhibition of Matrix Metalloproteases	4.13	0.289	-1.667
Neuregulin Signaling	1.99	0.149	-1.732
VEGF Signaling	2.25	0.155	-1.807
Oncostatin M Signaling	1.87	0.186	-1.89
Cyclins and Cell Cycle Regulation	2.2	0.162	-2.309
STAT3 Pathway	1.91	0.133	-2.496

Table S2. The combination index (CI) values of different drug combinations in GBM cancer cells

U87MG (72h, MTT assay)			T98G (72h, MTT assay)		
MPT0L145 (∝M)	Abemaciclib (∝M)	CI	MPT0L145 (∝M)	Abemaciclib (∝M)	CI
1.0	0.5	1.667	1.0	0.5	0.518
1.0	0.5	1.769	1.0	0.5	0.628
3.0	0.5	0.788	3.0	0.5	0.351
3.0	0.5	0.783	3.0	0.5	0.326
1.0	1.0	0.599	1.0	1.0	0.438
1.0	1.0	0.591	1.0	1.0	0.461
3.0	1.0	0.698	3.0	1.0	0.291
3.0	1.0	0.700	3.0	1.0	0.249

M059K (72h, MTT assay)		
MPT0L145 (∝M)	Abemaciclib (∝M)	CI
1.0	0.25	1.081
1.0	0.25	1.116
3.0	0.25	0.877
3.0	0.25	0.92
1.0	0.5	1.07
1.0	0.5	1.105
3.0	0.5	0.947
3.0	0.5	0.937
